# Fault Diagnosis for Rolling Bearings Using Optimized Variational Mode Decomposition and Resonance Demodulation

**DOI:** 10.3390/e22070739

**Published:** 2020-07-03

**Authors:** Chunguang Zhang, Yao Wang, Wu Deng

**Affiliations:** 1 School of Electronics and Information Engineering, Dalian Jiaotong University, Dalian 116028, China; zcg@djtu.edu.cn (C.Z.); yaow1211@163.com (Y.W.); 2 Traction Power State Key Laboratory of Southwest Jiaotong University, Chengdu 610031, China

**Keywords:** rolling bearings, fault diagnosis, VMD, resonance demodulation, parameter optimization, intrinsic mode function

## Abstract

It is difficult to extract the fault signal features of locomotive rolling bearings and the accuracy of fault diagnosis is low. In this paper, a novel fault diagnosis method based on the optimized variational mode decomposition (VMD) and resonance demodulation technology, namely GNVRFD, is proposed to realize the fault diagnosis of locomotive rolling bearings. In the proposed GNVRFD method, the genetic algorithm and nonlinear programming are combined to design a novel parameter optimization algorithm to adaptively optimize the two parameters of the VMD. Then the optimized VMD is employed to decompose the collected vibration signal into a series of intrinsic mode functions (IMFs), and the kurtosis value of each IMF is calculated, respectively. According to the principle of maximum value, two most sensitive IMF components are selected to reconstruct the vibration signal. The resonance demodulation technology is used to decompose the reconstructed vibration signal in order to obtain the envelope spectrum, and the fault frequency of locomotive rolling bearings is effectively obtained. Finally, the actual data of rolling bearings is selected to testify the effectiveness of the proposed GNVRFD method. The experiment results demonstrate that the proposed GNVRFD method can more accurately and effectively diagnose the fault of locomotive rolling bearings by comparing with other fault diagnosis methods.

## 1. Introduction

With the rapid development of the railway industry in China, the numbers of locomotives are continuously increasing, and the safe running of locomotives requires improvement. In order to guarantee the smooth and safe running of locomotives, the temperature detection method is fault prevention and fault alarm. Aiming at the defects of locomotive rolling bearings, the running status of locomotive rolling bearings is monitored by analyzing the vibration signals in the running process in order to diagnose the running status of the locomotive rolling bearings and ensure the safe running of the locomotives [1,2,3]. Therefore, it is very important to select an effective signal analysis method to deal with the collected vibration signal for obtaining the higher diagnosis accuracy.

In recent decades, various fault detection methods have been developed to realize the fault diagnosis of rotating machinery. These methods can be categorized into signal processing–fault diagnosis method, reasoning–fault diagnosis method, analytical model–fault diagnosis method and knowledge–fault diagnosis method [4,5,6,7,8,9,10]. The signal processing–fault diagnosis method is used to analyze the characteristics of measured vibration in time domain, frequency domain and time–frequency domain to determine the types and properties of various faults. The reasoning–fault diagnosis method does not depend on the mathematical model; on the basis of long-term practical experience and a large amount of fault information, this abstract knowledge in natural language is transformed into computer-understandable forms, such as production rules, frame representation, logic representation, and so on. The analytical model–fault diagnosis method uses the vibration parameters obtained by measurement to identify the simulation parameters, so as to determine the cause and location of the fault. This method needs to establish a more accurate mathematical model of the object to be diagnosed, while its advantage is that it has inherent sensitivity to unknown faults. The knowledge–fault diagnosis method realizes the fault diagnosis by processing the measured data.

In the development process of signal processing–fault diagnosis, the fault diagnosis methods can be divided into spectral analysis, cepstrum analysis, envelope analysis, and time–frequency analysis. Spectral analysis uses the characteristic spectrum and spectral intensity of substances in different spectral analysis methods for quantitative analysis. Cepstrum analysis can effectively extract and separate periodic abnormal vibration signal, but the cepstrum is also accompanied by harmonics and noise interference signal, which can easily cause misdiagnosis. Envelope analysis can decompose complex multi-component signals, but the anti-noise performance is insufficient, and it is mostly used as an auxiliary method for signal processing process. Time–frequency analysis is a research hotspot in the field of bearing fault diagnosis. The commonly used time–frequency analysis methods mainly include short-time Fourier transform, wavelet transform, S transform and so on [11].

The short-time Fourier transform is proposed to effectively identify the frequency and phase of sine wave in the local area of non-stationary signal, and its optimization processing [12,13,14,15]. Wavelet transform is a new transform analysis method, which inherits and develops the localization idea of short-time Fourier transform, and overcomes the shortcomings that the window size does not change with frequency [16,17,18]. The S-transform based on wavelet transform and short-time Fourier transform is proposed to eliminate the selection of window function, improve the defect of fixed window width, and maintain direct contact with the phase spectrum and original signal of each frequency component in time–frequency representation [19,20,21]. Empirical mode decomposition (EMD) is an adaptive data processing method, which is mainly used for nonlinear, non-stationary time series processing [22]. The integrated ensemble empirical mode decomposition (EEMD) is proposed to solve the problem of modal aliasing in the EMD. Local mean decomposition (LMD) is a new adaptive non-stationary signal processing method. Variational modal decomposition (VMD) method has a sufficient mathematical foundation, it can adaptively decompose the original signal to analyze the fault signal [23,24,25,26]. In addition, some new optimization methods are proposed to combine with signal processing methods in recent years [27,28,29,30,31,32,33,34,35,36,37,38].

In summary, these existing signal processing methods better achieve fault diagnosis of rolling bearings and obtain better diagnosis results. However, these signal processing methods have their own shortcomings and limitations in the fault diagnosis. The short-time Fourier transform is used to express the signal characteristics of a certain time through the signals in the time window, but its window function is invariable, so it has limitations on the analysis of abrupt signal and non-stationary signal. Wavelet transform overcomes the shortcomings of the window function invariance of short-time Fourier transform, and can analyze multi-resolution data, but it is difficult to choose its basis function [39,40,41]. Combining the advantages of the two methods, the resolution can adaptively be adjusted, so that the inverse transform is lossless and reversible, but the continuous signal can only be processed. The EMD is a new adaptive signal processing method in time domain, which is especially suitable for the analysis and processing of non-linear and non-stationary signals, but the processing results may have modal aliasing, end-point effects, over-envelope and under-envelope problems. The EEMD allows white noise to be added to the original signal to automatically distribute signals of different time scales to the appropriate reference scale. Due to the characteristics of zero-mean noise, after multiple averages, the noise will cancel each other and obtain the final signal processing result. The EEMD can effectively suppress the modal aliasing of the EMD, but the process is cumbersome and cannot effectively solve the problems of end-point effects. The LMD can effectively decompose complex non-stationary and multiple-component signals, but the calculation process of the decomposition is large, and some PF (product function) components are lacking references [42]. The VMD can effectively decompose non-stationary nonlinear signals, but its decomposition parameters need to be artificially set, which is prone to over-decomposition or under-decomposition phenomena [43]. In order to solve the problems of VMD, a 1.5-dimensional diagnostic method based on the optimized VMD with genetic algorithm is proposed for fault diagnosis [44]. However, a single genetic algorithm may easily fall into the parameter optimization process. A combination of VMD and resonance demodulation is proposed to perform signal processing. The signal processing results are straightforward, but there still exists the defect of parameter setting [45,46]. 

Resonance demodulation technology can extract the weak signal submerged in the background noise through the envelope analysis, then output a signal which eliminates the vibration signal interference, carries on the frequency spectrum analysis by using the fault characteristic frequency. It has the advantages of eliminating the external complex interference and effectively identifying the initial fault of rolling bearings. Therefore, in order to solve the shortcomings of the artificial setting parameters in the VMD, a novel parameter optimization algorithm based on genetic algorithm and nonlinear programming is designed to adaptively optimize the parameters of the VMD. Then a novel fault diagnosis method based on the optimized VMD and resonance demodulation technology, namely GNVRFD is proposed to realize the fault diagnosis of locomotive rolling bearings. The designed parameter optimization can effectively solve the variation mode. The optimized VMD and resonance demodulation technology can make the signal processing results clearer and eliminate the misdiagnosis caused by modal confusion, and scientifically and accurately assess the signal processing process of locomotive rolling bearings. In this paper, the novelty of this study is to design a novel parameter optimization algorithm and propose a novel fault diagnosis method.

The main contributions of this paper are described as follows:

- A novel fault diagnosis method (GNVRFD) proposed to effectively realize the fault diagnosis of locomotive rolling bearings.
- A novel parameter optimization algorithm for VMD is designed to adaptively optimize the parameters.
- The resonance demodulation technology is employed to decompose the reconstructed vibration signal in order to obtain the envelope spectrum and the fault frequency.
- The effectiveness of GNVRFD is extensively investigated by the actual data of rolling bearings.

## 2. Basic Methods

### 2.1. VMD 

The essence of the VMD is to determine the bandwidth and center frequency of each intrinsic mode function component by continuously searching for the optimal solution of the variation model, so that the sum of the bandwidths of the intrinsic mode function (IMF) components obtained by the decomposition is minimized, and the sum of the components is equal to the original signal ƒ, which can achieve efficient separation of the signal.

According to Dragomiretskiy’s introduction, when the VMD is used to process signals, it can be assumed that the original signal can be decomposed into k IMF components, each IMF component $u_{k}$ has a center frequency and a finite bandwidth, and the corresponding constrained variation model is described as follows [43].

(1) $$\underset{\left\{u_{k}\right\},\left\{\omega_{k}\right\}}{min}\left\{\overset{k}{\underset{k=1}{\sum }}\|\partial_{t}\left\{\left[\delta_{t}+\frac{j}{\pi t}\right]*\mu_{k}\left(t\right)\right\}e^{-j\omega_{k}t}{\|}_{2}^{2}\right\}$$

where $u_{k}=\left\{u_{1},u_{2},\ldots u_{k}\right\}$ is a set of modal component functions, the sum of them is the original function ƒ, $\partial_{t}$ is the partial derivative of time t, $\omega_{k}=\left\{\omega_{1},\omega_{2},\ldots \omega_{k}\right\}$ is the center frequency set of the modal component, $\delta_{t}$ is the unit pulse function, j is an imaginary unit,* is convolution.

(2) $$\xi \left(\left\{u_{k}\right\},\left\{\omega_{k}\right\},\lambda \right)=\alpha \overset{K}{\underset{k=1}{\sum }}\|\partial_{t}\left\{\left[\delta \left(t\right)+\frac{j}{\pi t}\right]*u_{k}\left(t\right)\right\}e^{-j\omega_{k}t}{\|}_{2}^{2}+\langle \lambda \left(t\right),f\left(t\right)-\overset{K}{\underset{k=1}{\sum }}u_{t}\left(t\right)\rangle$$

where ∂ represents the bandwidth parameter, λ(t) represents the Lagrangian multiplier, the value interval of k is 1 k. In the process of signal decomposition using VMD, the optimization of the decomposition result is realized by introducing the quadratic penalty factor ∂ and the Lagrangian multiplier λ(t), which can be obtained by Fourier transform. 

According to the model, the implementation process of the VMD is described as follows.

Step 1. Initialize $\left\{{\hat{u}}_{k}^{1}\right\}$,$\left\{\omega_{k}^{1}\right\}$,${\hat{\lambda }}^{1}$,n←0.
Step 2. Set the number of iterations n=n+1.
Step 3. For k = 1:K
  - Update function ${\hat{u}}_{k}$ for all ω≥0.
    (3) $${\hat{u}}_{k}^{n+1}\left(\omega \right)\leftarrow \frac{f\left(\omega \right)-\sum_{i=1,i<k}^{K}{\hat{u}}_{i}^{n+1}\left(\omega \right)-\sum_{i=1,i<k}^{K}{\hat{u}}_{i}^{n}\left(\omega \right)+\frac{{\hat{\lambda }}^{n}\left(\omega \right)}{2}}{1+2\alpha {\left(\omega -\omega_{k}^{n}\right)}^{2}}$$
  - Update function $\omega_{k}$.
    (4) $$\omega_{k}^{n+1}\leftarrow \frac{\int_{0}^{\infty }\omega {\left|{\hat{u}}_{k}^{n+1}\left(\omega \right)\right|}^{2}d\omega }{\int_{0}^{\infty }{\left|{\hat{u}}_{k}^{n+1}\left(\omega \right)\right|}^{2}d\omega }$$
Step 4. For all ω≥0, the following expression is executed.
  (5) $${\hat{\lambda }}^{n+1}\left(\omega \right)\leftarrow {\hat{\lambda }}^{n}\left(\omega \right)+\gamma \left[\hat{f}\left(\omega \right)-\overset{K}{\underset{k=1}{\sum }}{\hat{u}}_{k}^{n+1}\left(\omega \right)\right]$$
  where γ represents the noise margin parameter. When the signal contains more interference, in order to reduce the influence of the interference signal, set γ=0.
Step 5. Repeat Step 2~ Step 4 until the constraint ε is met.
  (6) $$\overset{K}{\underset{k=1}{\sum }}\|{\hat{u}}_{k}^{n+1}-{\hat{u}}_{k}^{n}{\|}_{2}^{2}/\|{\hat{u}}_{k}^{n}{\|}_{2}^{2}<\varepsilon$$

From the specific implementation process of the VMD, the VMD is relatively simple. The IMF components are searched iteratively in the frequency domain, and finally the Fourier inversion is solved in the time domain. The center frequency is the center of the power spectrum of the IMF component and is continuously re-estimated in the iterative process of the cyclic update.

### 2.2. Resonance Demodulation Technolgy

The theoretical basis of resonance demodulation lies in the modulation phenomenon, and the required fault information is separated by demodulation. In practical engineering applications, envelope demodulation is a very widely used demodulation method, which mainly uses the Hilbert transform to obtain the signal envelope and analyze the low frequency fault information [47,48,49].

Hilbert transform is used for a modulated signal $x_{m}$ model.

(7) $$x_{m}\left(t\right)=x_{m}\left[1+Acos\left(2\pi f_{n}t\right)\right]\sin\left(2\pi f_{z}t\right)$$

where $f_{n}$ is the low frequency modulation signal frequency, $f_{z}$ is the high frequency carrier frequency.

After Hilbert transform is executed, the following expression is obtained.

(8) $${\hat{x}}_{m}\left(t\right)=x_{m}\left[1+Acos\left(2\pi f_{n}t\right)\right]\sin\left(2\pi f_{z}\right)$$

where ${\hat{x}}_{m}\left(t\right)$ is obtained by transforming the original signal by 90°, and the analytic function is obtained as follows.

(9) $$z_{m}\left(t\right)=x_{m}\left(t\right)+j{\hat{x}}_{m}\left(t\right)$$

Thus, the envelope of the signal $z_{m}\left(t\right)$ can be obtained as follows.

(10) $$\left|z_{m}\left(t\right)\right|=\sqrt{x_{m}^{2}\left(t\right)+j{\hat{x}}_{m}^{2}\left(t\right)}=x_{m}\left|1+A_{m,1}cos\left(2\pi f_{n}t\right)\right|$$

To simplify the calculation, record the envelope of signal $\left|z_{m}\left(t\right)\right|$ as *E*, and perform Fourier expansion on the signal $f_{1}\left(t\right)=Ecos\left(\omega_{0}t\right)$.

(11) $$f_{2}\left(t\right)=\left|f_{1}\left(t\right)\right|=\frac{2E}{\pi }+\frac{4E}{\pi }cos\left(2\omega_{0}t\right)-\frac{4E}{15\pi }cos\left(4\omega_{0}t\right)+\frac{4E}{35\pi }cos\left(6\omega_{0}t\right)$$

It can be seen from the formula that after the envelope signal is obtained by Hilbert transform, the frequency domain transform is carried out, and finally the modulated low-frequency signal is obtained and presented in the frequency domain. Therefore, it can extract the effective information from the vibration signal of locomotive rolling bearings, and display it in the form of an oscillogram, and directly observe whether there is a potential fault in locomotive rolling bearings.

### 2.3. Information Entropy

Information entropy is a quantity of uncertainty, which is used to describe a source in thermodynamics [50]. In this paper, it is used as the fitness function of the parameter optimization algorithm to calibrate the solution interval. The entropy value of each component obtained by signal decomposition is used to reflect the sparse characteristics of the original signal. Certainty is directly proportional to its conversion formula as follows.

(12) $$p_{j}=a\left(j\right)/\overset{N}{\underset{j=1}{\sum }}a\left(j\right)$$

(13) $$E_{p}=-\overset{N}{\underset{j=1}{\sum }}p_{j}\log_{2}(p_{i})$$

where a(j) is the envelope signal obtained by the Hilbert demodulation of signal x(t), p(j) is the normalized form of signal a(j), and $E_{p}$ is an envelope entropy of the zero-mean signal x(t).

Taking the signal collection process of locomotive rolling bearings, when a rolling bearing fails, a relatively strong vibration will occur at the failure point, and periodic shock pulses will appear in the signal. When the collected vibration signal contains less noise, the signal waveform will be sparse. That is to say, the envelope entropy value of the signal is small. When the signal contains more interference information, the impact generated by the fault source will be masked. The fault features cannot be directly captured, and the signal waveform is dense. That is to say, the envelope entropy value of the original signal is large. The global minimum envelope entropy value is used as the parameter optimization criterion, and all components are screened to determine the parameter values in the VMD to achieve the parameter optimization.

## 3. Parameter Optimization of VMD 

In order to accurately decompose the vibration signal of locomotive rolling bearings by using the VMD, prevent the phenomenon of over-decomposition or under-decomposition, and obtain accurate bandwidth and center frequency for each component, it is necessary to optimize two parameters of the VMD. The combination of the modal number k and the penalty factor ∂ need to be solved. In the optimizing process of the VMD parameters, particle swarm optimization (PSO) and frog jumping algorithms are used to optimize the parameters of the variational modal decomposition method, respectively [51]. The PSO and frog leap algorithms are the same in principle, but the PSO algorithm easily falls into a local optimum and the search path is more complicated. The convergence speed of the frog leap algorithm is slower and may also fall into a local optimum and energy value. The two algorithms can only determine the number of modal components and the operation process is more complicated.

In the process of parameter optimization, the genetic algorithm is used to optimize the parameters of the VMD. The parameters of VMD are encoded into chromosomes instead of parameters, which reduces the limitation of function constraints. The starting point of the search process is a set of problem solutions, rather than a single individual. It has the characteristics of implicit parallel search through the iterative operation of chromosome selection, crossover, mutation and so on. The optimized solution of the parameters is obtained, which has strong global searching ability in the process of solving. Most of the classical nonlinear programming algorithms use the gradient descent method to solve the problem. It starts from an estimated value and searches for the minimum value of the nonlinear multivariate function under the constraint condition. The local search ability is strong, but the global search ability is weak.

Therefore, the genetic algorithm and nonlinear programming is combined to design a novel parameter optimization algorithm to optimize the parameters of the VMD. By cooperating with each other, the algorithm overcomes the shortcomings of the genetic algorithm, which may easily fall into premature convergence and local optimization. It also overcomes the shortcomings of complex calculation process and low accuracy of parameter optimization algorithms in this field. The new parameter optimization algorithm makes full use of the global search ability of the genetic algorithm and the local search ability of the nonlinear programming, reduces the possibility of the genetic algorithm falling into the local minimum, makes up for the shortcomings of local search ability of the genetic algorithm and the global search ability of the nonlinear programming. Therefore, the designed parameter optimization algorithm is used to optimize the parameters of the VMD in order to determine the optimized parameters of the VMD with the global optimal solution.

**Figure 1:**
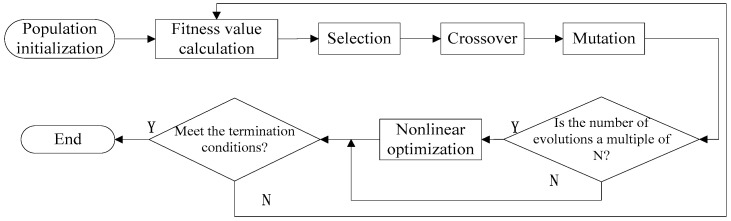
The flow of parameter optimization.

During the process of the parameter optimization, the genetic algorithm is used as the main body to improve its local search ability and search for the optimal value. The nonlinear programming is responsible for continuously adjusting the optimization range to reduce traps. The risk of a local optimum ensures that a global optimum is eventually found. A parameter optimization flow based on the combined genetic algorithm with nonlinear programming for the VMD is shown in Figure 1.

In the parameter optimization process, the genetic algorithm needs a fitness function, while the nonlinear programming needs a condition function to calibrate the solution interval. Therefore, the information entropy function is used to define the solution interval. It can well reflect the sparse characteristics of the signal. The transformed information entropy function of each component $u_{k}$ obtained by the VMD with parameters (k, ∂) is used as the fitness function of genetic algorithm. The conditional function is the global iterative optimization through the selection, mutation and crossover. Through the local search ability of nonlinear programming, the global optimal solution of the modal number k and the penalty factor ∂ can be solved.

In order to verify the feasibility of the designed parameter optimization algorithm, it is compared with the genetic algorithm. A simulation signal f(x) is constructed according to the characteristics of rolling bearing fault vibration signal in this paper. The expression of simulation signal is given in the formula (14).

(14) $$f\left(x\right)=-5sinx_{1}sinx_{2}sinx_{3}sinx_{4}sinx_{5}-sin5x_{1}sin5x_{2}sin5x_{3}sin5x_{4}sin5x_{5}+8$$

**Figure 2:**
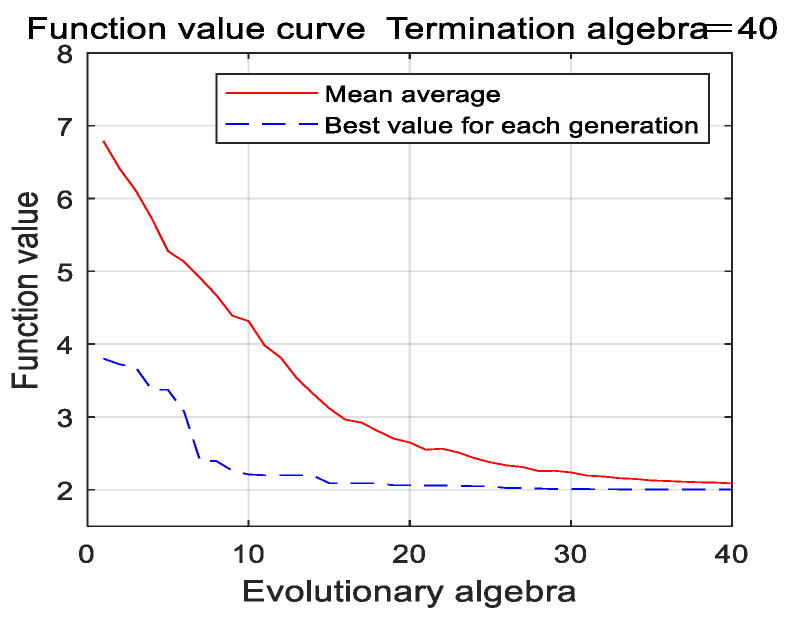
The iterative curve of optimization process using genetic algorithm.

**Figure 3:**
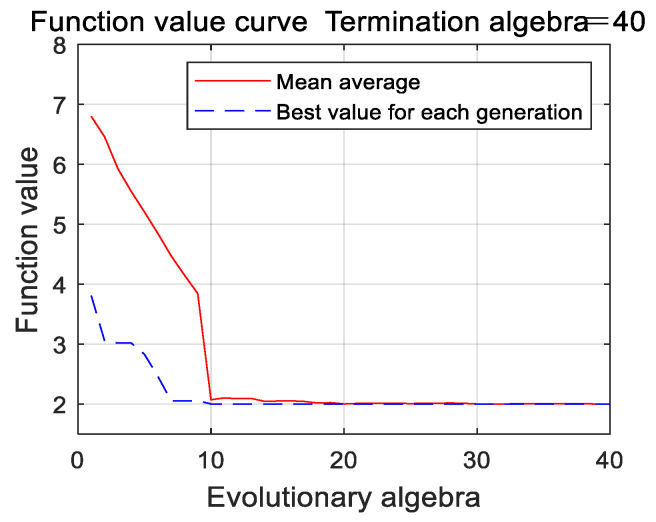
The iterative curve of optimization process using the parameter optimization algorithm.

In this simulation experiment, the expression (14) is regarded as the fitness function of the genetic algorithm and the objective function of the nonlinear programming. For the genetic algorithm, the number of iterations is 40, the population size is 150, the crossover probability is 0.6, and the mutation probability is 0.02, and the limited range of the nonlinear programming is 0–2.8724. The iterative curves of the optimization process are shown in Figure 2 and Figure 3.

As can be seen from the results of Figure 2 and Figure 3, the genetic algorithm falls into premature convergence, and the convergence speed decreases gradually. After 40 iterations are finished, the obtained result still has a large deviation with the optimal value. The designed parameter optimization algorithm obtains an approximate optimal solution at the 10th iteration under the same population size, its convergence speed is faster, the obtained optimal value is closer to the theoretical optimal value and the accuracy of parameter optimization is better than the traditional genetic algorithm. It can be concluded that the designed parameter optimization algorithm shows faster convergence speed, stronger global optimization ability and engineering practicability.

## 4. A Novel Fault Diagnosis Method (GNVRFD)

### 4.1. Novel Fault Diagnosis Method

When the locomotive rolling bearings are running, the collected vibration signal is a typical non-stationary and non-linear multiple component vibration signal. The vibration signal collected by the acceleration sensor is equivalent to the superposition of multiple vibration source signals, and there are many other vibration sources in the signal. In the process of signal analysis and fault diagnosis, the interference signal makes it more difficult to extract fault information. The theoretical basis of resonance demodulation is the modulation phenomenon, and the required fault information is separated by demodulation to further overcome the problems of modal confusion. If a complex multi-component signal can effectively reduce the noise and separate it into a single component signal of each vibration source, then an effective spectrum can greatly improve the accuracy of fault diagnosis. Therefore, the optimized VMD and resonance demodulation technology are integrated in order to propose a novel fault diagnosis method (GNVRFD), which is used to diagnose the fault of locomotive rolling bearings in this paper. The parameter optimization algorithm can make VMD results more accurate and practical. The optimized VMD can transform the complex non-linear and non-stationary vibration signal into a single component vibration signal. Rendering the noise clearer and easier to detect makes the vibration information of the fault point simpler to collect. The envelope spectrum of the reconstructed signal, which is composed of the sensitive components selected by the resonance modulation technology is selected to make the clear and accurate reconstructed signal. Therefore, the novelty of this study is to design a novel parameter optimization algorithm and propose a novel fault diagnosis method in this paper.

**Figure 4:**
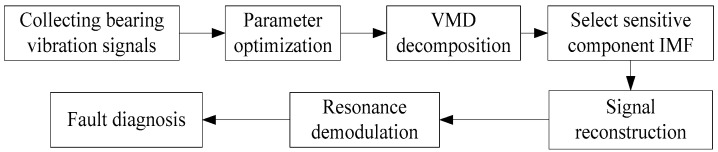
The fault diagnosis flow for locomotive rolling bearings.

The fault diagnosis process of the locomotive rolling bearings is shown in Figure 4.

### 4.2. Implementation Steps of the GNVRFD 

The implementation steps of the proposed GNVRFD method for locomotive rolling bearings are described in detail as follows.

Step 1. Initialize the parameters of the proposed GNVRFD method.
Step 2. The designed parameter optimization algorithm is used to determine the modal number *k* and the penalty factor *∂* of the VMD.
Step 3. The signal entropy function of the collected bearing vibration signal is used as an intermediate link to ensure the accuracy of the decomposition process.
Step 4. According to the determined decomposition parameter, the collected vibration signal of rolling bearings is decomposed by the optimized VMD, and each modal component is output as a kurtosis value.
Step 5. Among the obtained variables by the decomposition, two components with more sensitive kurtosis values are selected according to the maximum value principle to reconstruct the signal, and the reconstructed signal is subjected to resonance demodulation.
Step 6. The envelope spectrum obtained by resonance demodulation can directly obtain the peak value of the vibration frequency of the locomotive rolling bearings.
Step 7. Determine whether there is a potential fault of the locomotive rolling bearings and achieve the diagnosis of the locomotive rolling bearings.

## 5. Case Analysis

### 5.1. Data Source and Experimental Environment

**Figure 5:**
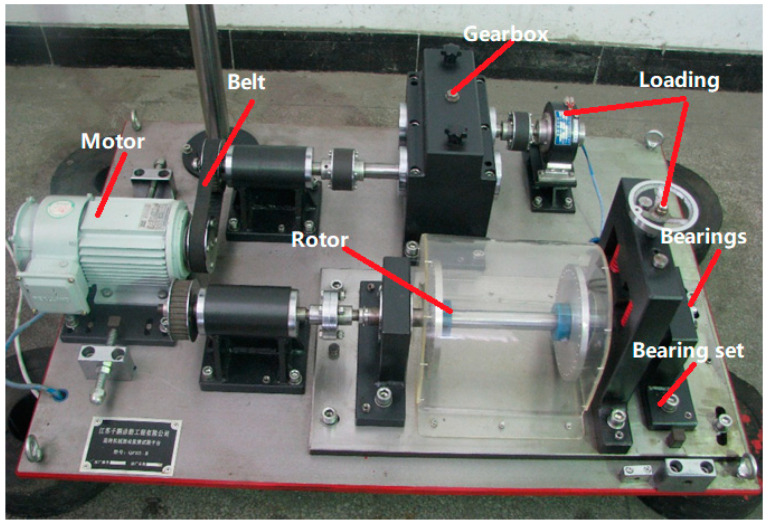
Experiment workbench.

In order to verify the proposed GNVRFD method, the potential fault of the locomotive rolling bearings can be accurately identified. The bearing fault workbench is used for the experiment, and the piezoelectric acceleration sensor is used to collect the vibration signal. The collected vibration signal is used to test the effectiveness of the proposed GNVRFD method in this paper. The deviation between the processing result and the theoretical fault value is verified whether the proposed GNVRFD method can accurately identify the potential fault of the locomotive rolling bearings. The test bench in this experiment is a bearing vibration measuring instrument based on QPZZ-II system. The QPZZ-II system can use the acceleration sensor, torque sensor, temperature sensor, eddy current sensor, photoelectric sensor and other electronic components to collect the real-time signals of the rotating machinery. It can quickly simulate various states and vibrations of rotating machinery, and can also carry out comparative analysis and diagnosis of various states. The number of the collected data points is 2000, the speed of the rolling bearings is 900 r/min, and the sampling frequency is 25k Hz. The experiment workbench is shown in Figure 5.

The specific parameters of the experiment equipment are shown in Table 1. According to the parameters of rolling bearings, we can calculate the theoretical fault frequency of rolling bearing inner ring. 

The calculation formula of fault frequency of rolling bearing inner ring is given as follows.

BPFI = r/60*1/2*z*[1 + (d/D)*cosα] BPFI = 900/60*1/2*13*[1 + (24/39)*cos0] = 157.5 Hz(15)

**Figure 6:**
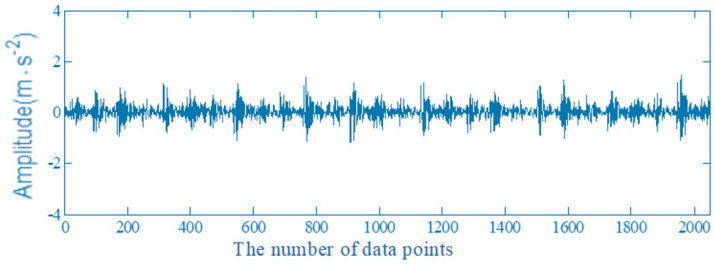
The time domain waveform of vibration signal of a rolling bearing inner ring.

According to the equation (15), the theoretical value of the inner ring fault is 157.5 Hz. The time domain waveform of the collected vibration signal of rolling bearing inner ring is shown in Figure 6. In the time domain waveform of vibration signal, Y-axis is the amplitude (acceleration), the unit is $m\cdot s^{-2}$. X-axis is the number of the collected data points.

### 5.2. Experimental Result and Analysis

After the data acquisition of rolling bearings is completed, the designed parameter optimization algorithm is used to determine the two parameters of the VMD, which is mainly completed by the genetic algorithm and nonlinear programming. The minimum envelope entropy value obtained by the parameter optimization algorithm can be determined. The number of modes of the VMD is k = 5, and the penalty factor is ∂ = 1817. Then the collected vibration signal of rolling bearing is decomposed and the components are output in the form of kurtosis values, and the kurtosis values of each components are obtained in Table 2.

**Figure 7:**
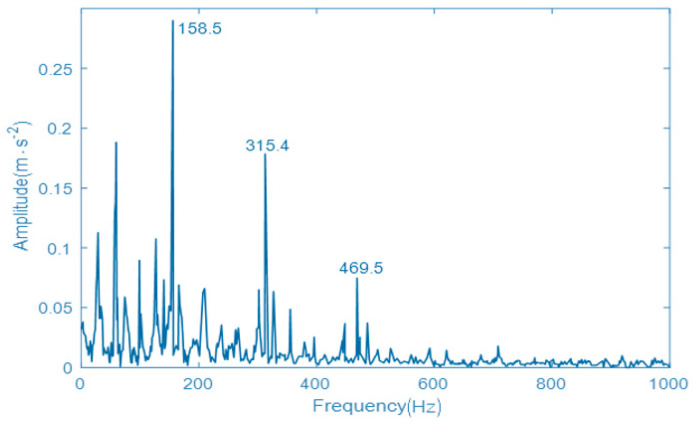
The envelope spectrum of fault signal using the variational mode decomposition (VMD) with designed parameter optimization algorithm.

By comparing the components, IMF3 and IMF5 have the largest kurtosis value. According to the principle of maximum kurtosis value, the two components of IMF3 and IMF5 are reconstructed, and the reconstructed signal is resonantly demodulated to obtain the envelope spectrum by using resonance demodulation technology. The envelope spectrum of fault signal using the VMD with the designed parameter optimization algorithm is shown in Figure 7. In the obtained vibration signal spectrum, the Y-axis is the amplitude (acceleration), the unit is $m\cdot s^{-2}$. X-axis is the frequency, the unit is Hz.

As can be seen from Figure 7, the spectral peak at 50 Hz is the natural vibration frequency of locomotive rolling bearings, which is only used as a reference in the fault diagnosis. When the frequency is 158.5 Hz, a spectral peak appears in the envelope spectrum. Although the error between the actual peak frequency and the theoretical fault frequency is very small, it can be ignored because of the geometric error and assembly error of the rolling bearing. Therefore, it can be considered that the envelope spectrum of the signal reflects the fault characteristics of the rolling bearing inner ring. It can also be determined that there is a certain degree of corrosion or wear of the rolling bearing inner ring during operation. In addition, due to the running environment and other reasons, when the locomotive rolling bearing fails, the damage degree of rolling bearings will increase exponentially, and its vibration frequency will gradually increase. The appeared faults are more severe. Therefore, the normal state and the fault state of a rolling bearing are only simulated to verify the availability and accuracy of the proposed fault diagnosis method.

Because the experiment only collects the vibration data of rolling bearing inner ring, a better diagnosis result is obtained. According to the calculation formula of rolling bearing fault characteristic frequency, it can be known that when a rolling bearing fails at different positions, the fault characteristic frequency is different. Therefore, the envelope spectrum and fault characteristic frequency are obtained by using the signal processing method. In contrast, the rolling bearing is not only faulty, but also the fault detection can be achieved.

### 5.3. Comparison and Analysis

**Figure 8:**
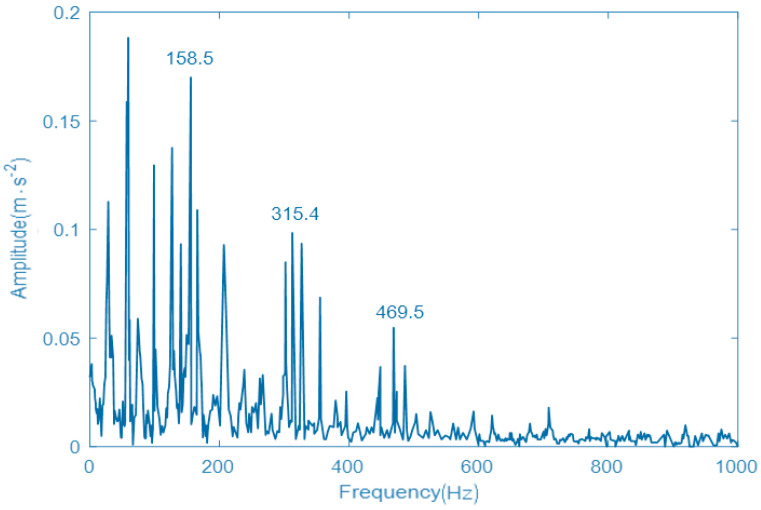
The envelope spectrum of vibration signal using optimized empirical mode decomposition (EMD).

**Figure 9:**
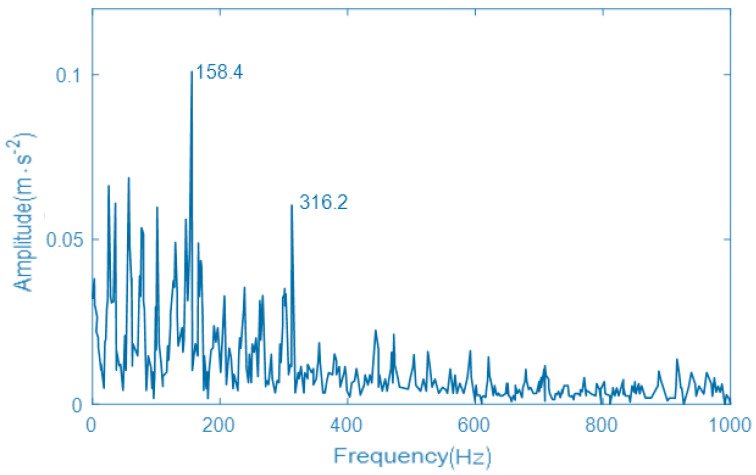
The envelope spectrum of vibration signal using VMD with particle swarm optimization (PSO) algorithm.

**Figure 10:**
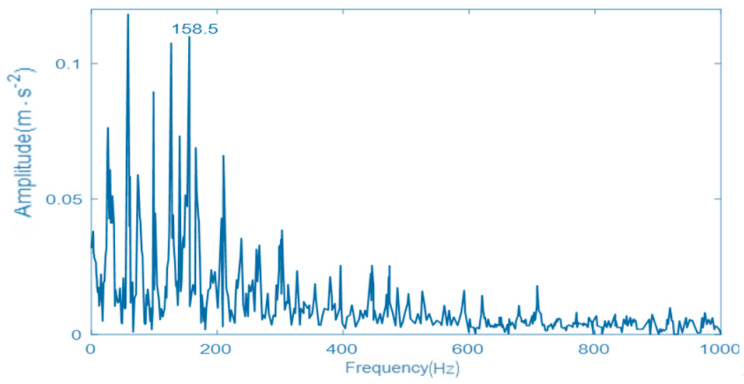
The envelope spectrum of vibration signal using resonance demodulation technology.

In order to further prove the effectiveness of the proposed GNVRFD method, the fault diagnosis method based on the optimized EMD, the fault diagnosis method based on the VMD with PSO algorithm, and the fault diagnosis method based on resonance demodulation technology are selected herein. The vibration signal and parameter setting are the same as the Section 5.2. The obtained envelope spectrums of vibration signals, by using three different fault diagnosis methods, are shown in Figure 8, Figure 9, and Figure 10. In the obtained vibration signal spectrum, the Y-axis is the amplitude(acceleration), the unit is $m\cdot s^{-2}$. X-axis is the frequency, the unit is Hz.

As can be seen from Figure 8, the EMD decomposes the fault signal with poor performance. Although the fault point and part of the multiplier can be observed, there will be more interference signals nearby the fault point, the double frequency and triple frequency. It is difficult to distinguish the specific fault characteristics, and the modulation characteristics of the vibration signal of a rolling bearing inner ring are not demodulated, and there is still a certain modal aliasing phenomenon, which seriously affects the state of a rolling bearing. As can be seen from Figure 9, although the fault diagnosis method can also obtain the fault frequency and double frequency, the processing effect of vibration signal of the rolling bearing inner ring is not very satisfactory, the natural frequency of the rolling bearing inner ring is submerged, and the envelope spectrum has more peaks and lower vibration energy, which has a certain impact on the result accuracy of fault diagnosis. As can be seen from Figure 10, the envelope spectrum of the vibration signal of a rolling bearing inner ring obtained by resonance demodulation technology is confusing. The strong noise completely submerges the periodic pulses in the fault signal. The envelope spectrum does not appear to adhere to any rule. There is a small peak near the fault, but it is not obvious. Therefore, it is impossible to diagnose the inner race fault of the rolling bearing accurately.

By comparing the envelope spectrum of the vibration signal of a rolling bearing inner ring obtained by different fault diagnosis methods, it can be seen that the proposed GNVRFD method effectively overcomes the shortcomings of modal confusion by comparing with other methods, reduces the generation of pseudo components and takes on better noise suppression. Under the strong noise interference, the proposed GNVRFD method can successfully and effectively extract the relevant fault information, and clearly display the relevant fault information in the envelope spectrum, so as to intuitively and accurately determine whether there is a potential fault in the running process. The purpose is to realize the detection of locomotive rolling bearings and ensure the safe and stable running of locomotives.

## 6. Conclusions

In this paper, a novel fault diagnosis method based on the optimized VMD and resonance demodulation technology, namely GNVRFD, is proposed to realize fault diagnosis of locomotive rolling bearings. In the GNVRFD method, the genetic algorithm and nonlinear programming are combined to optimize the two parameters of the VMD, and the optimized VMD method is used to decompose the collected vibration signal into a series of IMFs, and the kurtosis value of each IMF is calculated, respectively. The two most sensitive IMF components are selected for signal reconstruction, Then the reconstruction signal is decomposed by the resonance demodulation technology to obtain the envelope spectrum and realize the fault diagnosis of locomotive rolling bearings. The simulation experiment and comparative results show that the proposed GNVRFD method has an obvious advantage by combining the traditional envelope demodulation method and EMD method. In the decomposition process of the vibration signal, the proposed GNVRFD method can effectively suppress noise interference and end effect and modal aliasing. Moreover, the fault frequency is more accurately extracted from the vibration signal with strong noise, which can effectively diagnose the potential fault of the locomotive rolling bearings. The proposed GNVRFD method can effectively ensure the safe running of the locomotive. The rapid development of the railway industry and China’s economy is of great significance.

## Figures and Tables

**Table 1:** Test parameters of rolling bearings.

Model	Pitch	Inside Diameter	Number of Rollers	Roller Diameter	Contact Angle
N205EM	D(mm)	d(mm)	z	d2(mm)	α
Cylindrical Roller Bearings	39	24	13	7.5	0

**Table 2:** Modality values of various modal components.

IMF	IMF1	IMF2	IMF3	IMF4	IMF5
Kurtosis	1.2132	2.8463	3.7825	1.6542	4.6253
